# Research Progress of Biosensing Technology in the Detection of Creatine Kinase Isoenzyme MB

**DOI:** 10.3390/mi16101111

**Published:** 2025-09-29

**Authors:** Qixing Pan, Mingliang Jin, Qi Liang, Fengxia Lin, Yechu Dai, Zhenping Liu, Lingling Shui, Jiamei Chen

**Affiliations:** 1 Guangdong Basic Research Center of Excellence for Structure and Fundamental Interactions of Matter, Guangdong Provincial Key Laboratory of Nanophotonic Functional Materials and Devices, School of Optoelectronic Science and Engineering, South China Normal University, Guangzhou 510006, China; 2024022468@m.scnu.edu.cn (Q.P.); jinml@m.scnu.edu.cn (M.J.); liuzhenping@m.scnu.edu.cn (Z.L.); 2 The Seventh Clinical College of Guangzhou University of Chinese Medicine, Guangzhou University of Chinese Medicine, Shenzhen 518133, China; liangqi70@gzucm.edu.cn (Q.L.); szlinfx@163.com (F.L.); daiyechu@163.com (Y.D.); 3 Department of Pharmacy, Shenzhen Bao’an Chinese Medicine Hospital, Guangzhou University of Chinese Medicine, Shenzhen 518133, China

**Keywords:** biosensor, acute myocardial infarction, creatine kinase isoenzyme MB

## Abstract

Although significant progress has been made in the global medical level, cardiovascular diseases still pose a serious threat to human life and health. Among many cardiovascular diseases, acute myocardial infarction (AMI) is particularly severe. If not treated in a timely manner, it may lead to serious consequences such as cardiac arrest and sudden death. Early diagnosis of myocardial infarction (MI) is an important means of preventing and controlling the mortality rate of AMI. Creatine kinase isoenzyme (CK-MB) is a key biomarker of MI. It rises rapidly within 2 h after myocardial injury, reaches its peak at 24 h, and returns to normal at 72 h. Furthermore, CK-MB has a high specificity in monitoring secondary MI. Therefore, the early, real-time, and accurate detection of CK-MB is of great significance for the prevention, diagnosis, and prognosis of AMI. Conventional CK-MB detection methods have problems such as false positive elevation, large blood sampling volume, long time consumption, and complex operation, making it difficult to meet the needs of point-of-care testing (POCT). Biosensor technology, with its low cost, high sensitivity, and portability, offers a promising solution for point-of-care CK-MB testing, thereby greatly aiding AMI diagnosis.

## 1. Introduction

Cardiovascular diseases (CVDs) are the leading cause of death globally, with acute myocardial infarction (AMI) being the primary etiology contributing to CVD-related mortality [1,2,3]. AMI results from the sudden occlusion of a coronary artery, which precipitates irreversible myocardial ischemia and hypoxia, leading to severe, permanent cardiac muscle damage and posing a life-threatening risk to patients [4,5,6]. Globally, CVDs are responsible for approximately 30% of all deaths. In Europe alone, AMI is the leading single cause of mortality, claiming the lives of approximately 862,000 males (19% of total deaths) and 877,000 females (20% of total deaths) annually [7]. Furthermore, the 2023 China Cardiovascular Disease Report indicates a rising AMI mortality rate in China, with over one million new cases now reported each year [8].

The clinical diagnosis of AMI has traditionally relied on patient symptomatology, medical history, and characteristic changes on a standard electrocardiogram (ECG). However, clinical data indicate that approximately one-third of AMI patients do not present with typical symptoms in the early stages, and nearly half exhibit non-specific ECG changes [9]. To mitigate the risk of missed diagnoses, serological detection of cardiac biomarkers has become a cornerstone of modern AMI diagnostics. Key biomarkers include creatine kinase-MB (CK-MB), cardiac troponin T (cTnT), cardiac troponin I (cTnI), and myoglobin (Myo) [10,11,12]. Among these, CK-MB is a critical indicator of myocardial injury. Serum CK-MB levels typically become elevated within 4 to 6 h following AMI onset, peaking between 18 and 24 h at concentrations ranging from 39 ng/mL to 185 ng/mL [13]. Crucially, CK-MB demonstrates high specificity for monitoring recurrent or secondary myocardial injury [14]. Consequently, the early, real-time, and accurate detection of CK-MB is paramount for the effective prevention, diagnosis, and prognosis of AMI.

Current clinical methods for CK-MB detection primarily involve enzyme activity and enzyme mass assays. While the enzyme activity assay is widely used due to its operational simplicity and low cost, it is susceptible to interference from other creatine kinase isoenzymes, such as CK-BB from neurological tissue. This can lead to falsely elevated CK-MB activity in the presence of hemolysis or macro-CK, presenting diagnostic challenges of AMI [15,16,17]. In contrast, enzyme mass assays, like chemiluminescent immunoassays, leverage specific antigen–antibody binding, overcoming the limitations of activity-based methods. However, these assays require specialized instrumentation, trained personnel, and multi-step procedures (e.g., immobilization, incubation, washing), which can introduce significant delays to diagnosis and treatment. Therefore, there is a pressing need to develop novel, rapid, and accurate technologies for CK-MB detection.

Given the limitations of conventional methods, biosensor technology has emerged as a prominent area of research. As defined by the International Union of Pure and Applied Chemistry (IUPAC), a biosensor is a device that integrates a biological recognition element with a physicochemical transducer to detect a target analyte [18,19,20]. As a product of interdisciplinary fusion across chemistry, biology, physics, and engineering, biosensors provide powerful tools for analyzing and quantifying molecular interactions, rendering them indispensable for medical diagnostics [21,22,23]. For CK-MB detection specifically, biosensors offer compelling advantages, including low cost, high sensitivity, operational convenience, and high selectivity. These attributes position them as a promising solution for the real-time detection of CK-MB, offering robust support for the early diagnosis and timely management of AMI.

**Figure 1:**
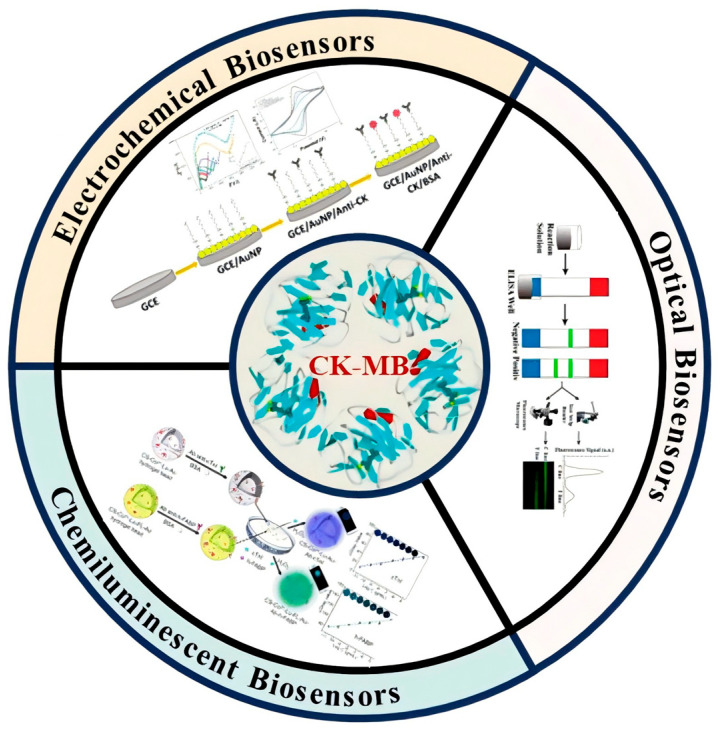
Biosensor technology of CK-MB.

As shown in Figure 1, this review aims to systematically evaluate recent advancements in biosensing technologies for CK-MB detection. It could provide insights into innovative methodologies that can support the development of next-generation diagnostic tools for the precise and timely management of AMI.

## 2. Biosensors for CK-MB Detection

Significant progress has been made in the development of biosensors for CK-MB detection, driven by their potential to overcome the limitations of traditional assays. These biosensors can be broadly classified based on two key features: the molecular recognition element and the signal transduction mechanism. Based on recognition elements, they are categorized as immunosensors, aptasensors, or enzyme-based biosensors, which utilize antibodies, nucleic acid aptamers, or enzymes, respectively, for highly selective target binding [24,25,26]. Based on signal transduction, they are classified as electrochemical, electrochemiluminescent, fluorescent, or optical biosensors, among others [27,28,29,30,31]. This diversity in design enables a wide array of detection strategies, enhancing both the sensitivity and accuracy of the measurements. This section will review the various types of biosensors for CK-MB detection, categorized by their transduction mechanism, with their analytical performance summarized in Table 1.

### 2.1. Electrochemical Biosensors

**Figure 2:**
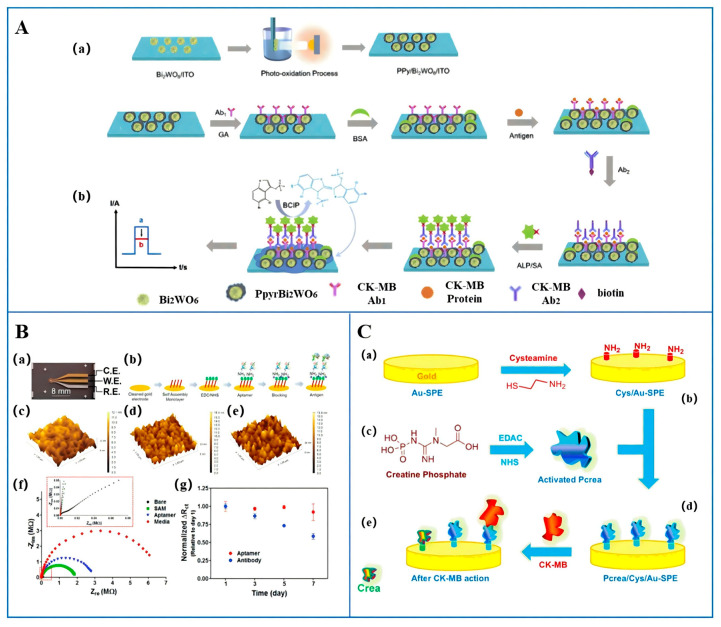
Electrochemical biosensors for the detection of CK-MB. (**A**) An immunoelectrochemical biosensor. (**a**) Preparation of organic−inorganic heterojunction of PPy@Bi_2_WO_6_ and (**b**) application for the PEC immunoassay of CK-MB via an enzymatic BCP process [33]; (**B**) an aptamer electrochemical biosensor. (**a**) photograph of the microfabricated electrode set, (**b**) schematic diagram of immobilization steps of aptamers onto the microelectrode, AFM surface characterization of (**c**) bare Au WE electrode, (**d**) antibody-, and (**e**) aptamer-functionalized microelectrode, (**f**) EIS measurements after immobilization of aptamer and subsequent molecules onto bare microelectrode, (**g**) Normalized ΔRct over time showing the denaturation of antibody and aptamer biosensors [39]; (**C**) an enzyme–substrate electrochemical biosensor. (**a**) bare gold of the Au-SPE, (**b**) amine monolayer formation, (**c**) activation of creatine phosphate, (**d**) creatine immobilization on the Cys/Au-SPE and (**e**) reaction of CK-MB [40] (Copyright [9,15,16]).

Electrochemical biosensors detect analytes by leveraging changes in electrochemical signals triggered by the interaction between the analyte and recognition elements on the electrode surface [32]. Immunological electrochemical sensors utilize the specific binding between CK-MB and corresponding antibodies to generate electrochemical signals, thereby enabling quantitative detection of CK-MB. As shown in Figure 2A, Zhu et al. [33] enhanced the detection performance by employing a polypyrrole@Bi_2_WO_6_ tungstate enhanced organic–inorganic heterojunction-modified electrode surface, combined with strategies such as antibody immobilization, antigen capture, secondary antibody binding, and alkaline phosphatase (ALP) labeling. The biocatalytic precipitation generated by the enzymatic reaction produced a precipitation-dependent inhibition of the photocurrent signal. The limit of detection (LOD) was 0.16 ng/mL, with a linear range of 0.5–2000 ng/mL and an *R*^2^ of 0.995. Cen et al. [34] employed an environmentally friendly one-step aqueous-phase method, assisted by 4-aminopyridine, to synthesize ultrathin gold–palladium–copper alloy (AuPdCu) nanowire networks (NWNs) on the electrode surface. This method is simple, seed-free, template-free, and surfactant-free, and does not involve the use of toxic organic solvents or special equipment during the preparation process. The binding of CK-MB to its antibody could trigger the generation of electrochemical signals. The ultrathin AuPdCu/NWNs possess a large specific surface area, high electronic conductivity, and good biocompatibility, and can efficiently catalyze the reduction reaction of hydrogen peroxide (H_2_O_2_), thereby enhancing the detection response. This enabled the ultra-sensitive detection of CK-MB with a LOD of 0.88 pg/mL and a linear range of 0.001–2000 ng/mL. De Lima et al. [35] reported a novel biodegradable composite electrode with a matrix of Poly(3-hydroxybutyrate-co-3hydroxyvalerate) and Poly(butylene adipate-co-terephthalate) (PHBV:Ecoflex), compounded with graphite microparticles. The graphite microparticles were uniformly distributed within the polymer blend structure, characterized by low defects, high conductivity, and high electron transfer kinetics. The developed biosensor has good flexibility and is suitable for point-of-care testing applications. The electrode was modified using the conductive polymers polyethyleneimine (PEI) and Pcrea, and CK-MB antibodies were also immobilized on the electrode surface. The binding of CK-MB to the Pcrea-functionalized electrode surface triggers an enzymatic reaction, generating an electrochemical signal. Using electrochemical impedance spectroscopy (EIS) for detection with a LOD of 0.26 ng/mL, a limit of quantitation (LOQ) of 0.88 ng/mL, and a linear range of 5.0–100.0 ng/mL, Demirbakan [36] developed an immunosensor system for measuring creatine kinase (CK) levels. This device employs disposable indium tin oxide-polyethylene terephthalate (ITO-PET) electrodes and incorporates various electrochemical techniques. The novelty of this study lies in the covalent modification of the ITO-PET surface, which has been rendered conductive with gold nanoparticles (AuNPs) using an 11-mercaptoundecanoic acid (11-MuA) reagent. This modification enables a wide dynamic range of determination, allowing for precise measurements at the picogram level. In addition to the fundamental technique of electrochemical impedance spectroscopy (EIS), the immunosensor system integrates multiple techniques such as CV, SWV, and SFI. The measurement results obtained are stable and consistent, demonstrating the system’s high sensitivity and stability. The proposed immunosensor system exhibits a broad dynamic range (0.1–100 pg/mL), with a limit of detection of 0.018 pg/mL and a limit of quantification of 0.039 pg/mL. In the context of literature reviews, this study presents a notable advancement in the field of CK detection. Zhong et al. [37] developed and characterized an electrochemical immunosensor based on gold nanoparticles (AuNPs) for the sensitive and specific detection of CK, a crucial cardiac marker. In the sensor design, AuNPs were meticulously optimized and assembled onto the electrode surface, followed by the immobilization of anti-CK antibodies. By leveraging the advantages of electrochemical detection and nanomaterial-enhanced sensing, this platform is capable of delivering superior analytical performance. The sensor achieved optimal performance under conditions of pH 7.4, with an antibody concentration of 100 μg/mL and an incubation time of 45 min. The sensor exhibited a sensitivity of 152.6 Ω/(ng/mL) and maintained its response in the presence of common interfering substances, with a maximum signal deviation of 7.2%. Testing with spiked human serum samples yielded recovery rates ranging from 96.5% to 103.8%.

Compared to immunosensors, aptasensors exhibit significant advantages in terms of environmental stability, convenience of artificial synthesis, and storage performance [38]. Shin et al. [39] developed a microfluidic aptamer-based electrochemical biosensor for CK-MB detection (Figure 2B). The platform integrates a three-electrode system (RE/CE/WE) with gold (Au) electrodes for stability. Aptamers were immobilized via Au-S bonding, carbodiimide coupling, and streptavidin–biotin reactions. CK-MB concentration was quantified by monitoring charge transfer resistance changes using EIS. The biosensor achieved a LOD of 2.4 pg/mL and linear range of 0.01–100 ng/mL. Cardiac chip and doxorubicin-induced injury experiments confirmed dose-dependent detection of trace CK-MB secreted by heart-like organs, with results correlating with tissue beating and cell viability data. This enables integration into organ-on-a-chip platforms for real-time biomarker monitoring.

Enzyme–substrate electrochemical sensors utilize the enzymatic reaction between CK-MB and its substrate to generate an electrochemical signal. As illustrated in Figure 2C, Felismina et al. [40] developed a self-assembled modified gold-based printed electrode (Au-SPE) for the detection of CK-MB. This method involved the amination of Au-SPE using cysteamine (Cys) followed by the immobilization of creatine phosphate (Pcrea) on the electrode surface through self-assembled monolayer technology. Due to the electroactivity of Pcrea at low potentials, CK-MB catalyzed its reaction, leading to changes in the electrical response of the biosensor, thereby enabling the detection of CK-MB. The LOD achieved using square wave voltammetry (SWV)was 0.11 μg/mL with a linear range of 0.19–28.8 μg/mL.

In summary, electrochemical biosensing technologies provide a diverse range of high-sensitivity and convenient solutions for CK-MB detection. However, their translation into practical applications is hindered by several technical bottlenecks. The influence of background noise and interfering species on signal accuracy remains a significant challenge, while the long-term stability and reproducibility of the sensors require substantial improvement [41]. Particularly for analysis in complex biological matrices, enhancing the sensitivity and selectivity of these sensors remains a critical research objective that must be addressed to realize their full clinical potential [42].

### 2.2. Chemiluminescent Biosensors

**Figure 3:**
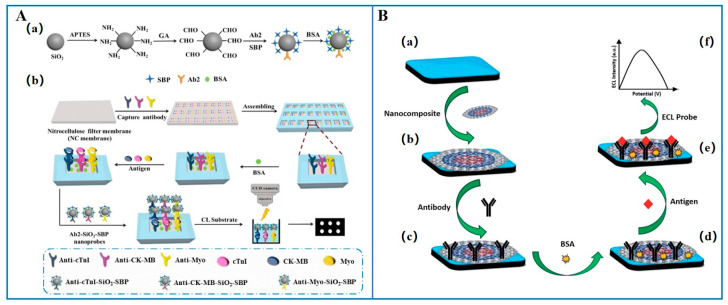
Chemiluminescent biosensors for the detection of CK-MB. (**A**) Chemiluminescent biosensor. (**a**) the preparation of Ab_2_-SiO_2_-SBP and (**b**) the general preparation procedures of immunosensing biochip and CL imaging immunoassay procedure [45]; (**B**) electrochemiluminescent biosensor. (**a**) SWCNTs-SPE, (**b**) CNOs/Fe_3_O_4_/AuNPs/CS modified SPE, (**c**) anti-CKMB spiked immunosensor, (**d**) BSA blocked immunosensor, (**e**) immunosensor spiked with the target antigen (CK-MB), (**f**) ECL signal [48] (Copyright [19,22]).

Chemiluminescence (CL) refers to the emission of light resulting from a chemical reaction between a labeled enzyme and its substrate. Chemiluminescence biosensors measure specific detection data by quantifying the emitted photons, and since they do not require an external excitation light source, they avoid interference from scattered light, consequently exhibiting high sensitivity [43]. Wang et al. [44] proposed a CK-MB detection method that combined magnetic separation with chemiluminescence. This method utilizes CK-MB monoclonal antibodies labeled with fluorescein isothiocyanate (FITC) and N-(4-aminobutyl)-N-ethylisoluminol (ABEI), as well as magnetic microbeads functionalized with anti-FITC antibodies. After simple steps such as magnetic separation, removal of the supernatant, and addition of chemiluminescence reaction reagents, the chemiluminescence intensity was used as the detection signal. The method showed good linearity when the CK-MB concentration is within the range of 0.0–500.0 ng/mL, with a detection limit of 0.296 ng/mL. As illustrated in Figure 3A, Zhao et al. [45] constructed an immunosensor biochip using an anionic soybean peroxidase (SBP)-functionalized nanoprobe and chemiluminescence imaging for simultaneous detection of three AMI biomarkers (cTnI, CK-MB, Myo). The nanoprobe (Ab2-SiO_2_-SBP) combined SBP and detection antibodies on silica nanoparticles to boost SBP loading and sensitivity. The biochip featured a 3 × 8 well array with capture antibodies and active sites per well. Target proteins triggered a CL signal via sandwich immunoassay, enabling single-well detection. For CK-MB, it showed a linear range of 0.2–120 ng/mL, LOD of 0.067 ng/mL, and 92–106.4% recovery in serum samples. Yin et al. [46] developed a snail-shaped microfluidic chip (SMC) based on the principle of CL for the multiplex detection of CK-MB, Myo, and cTnI. The SMC consisted of a three-layer structure, including a channel layer with mixers and reaction zones, a reaction layer coated with capture antibodies, and a base layer. The opening or closing of microchannels was achieved by controlling the downward movement of a press-type mechanical valve, and CL was used as the signal readout method. The chip had a LOD of 1.37 ng/mL for CK-MB and exhibited a good linearity in the range of 0.08–10.24 ng/mL. Additionally, this biosensor demonstrated excellent specificity and anti-interference capabilities. In clinical sample testing, the results were consistent with those obtained using hospital ELISA kits, showing good correlation for both healthy and patient samples.

In addition to traditional CL biosensors, electrochemiluminescence (ECL) biosensors have also been applied to the detection of CK-MB. ECL refers to the luminescence process in which substances generated on the electrode surface undergo electron transfer reactions to form excited states that emit light [47]. Adhikari et al. [48] constructed a label-free ECL immunosensor based on a novel nanocomposite-modified printed electrode for the ultrasensitive detection of CK-MB. As illustrated in Figure 3B, the nanocomposite was composed of carbon nano-onions (CNOs), gold nanoparticles (AuNPs), iron oxide (Fe_3_O_4_), and chitosan (CS). The nanocomposite was deposited on the working electrode, and CK-MB antibodies were immobilized on the electrode surface. Tris(2,2′-bipyridyl)ruthenium(II) chloride ([Ru(bpy)_3_]^2+^Cl) was used as the luminophore with tri-n-propylamine (TPrA) selected as the co-reactant due to its immobility in water and luminescent properties. After the immunoreaction between CK-MB and the antibody, an ECL signal was generated, enabling the quantitative detection of CK-MB. The developed biosensor showed a wide linear range (10 ng/mL to 50 fg/mL) and an extreme LOD (5 fg/mL). In real sample detection, the recovery rate ranged from 98% to 103%, demonstrating great potential for clinical detection of CK-MB.

In summary, CL biosensors leverage enzyme–substrate reactions to generate a light signal, which is quantified to determine analyte concentration. This method obviates the need for an external light source, thereby minimizing background interference from scattered light and enabling high sensitivity, with some systems achieving detection limits in the fg/mL range. A key sub-category, ECL, combines electrochemical and luminescent principles to generate light via electron transfer reactions at an electrode surface, further enhancing sensitivity. Overall, these sensors demonstrate robust clinical applicability and anti-interference capabilities for CK-MB detection. Nevertheless, challenges persist. CL systems require careful optimization of reagent storage conditions (e.g., pH, temperature) to ensure stability. For ECL sensors, the long-term stability of electrode modifications, such as nanomaterial coatings, is a critical concern. Furthermore, the linear dynamic range and robustness against matrix effects in clinical samples require further refinement for some platforms. Despite these challenges, this technology holds significant promise for advanced CK-MB detection.

### 2.3. Optical Biosensors

**Figure 4:**
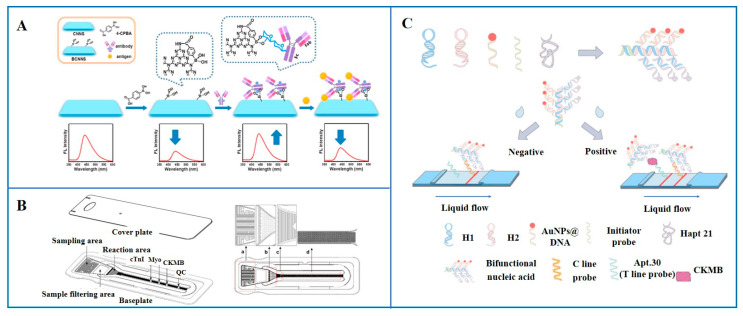
Fluorescent biosensors for the detection of CK-MB. (**A**) Fluorescent immune-biosensor based on boronic acid-modified carbon nitride nanosheets [50]; (**B**) fluorescent biosensor based on a microfluidic chip-based immunochromatographic strategy. (a) the sampling area, (b) the reaction area, (c) the time-controlled valve, and (d) the detection area [51]; (**C**) fluorescent biosensor based on aptamer [54] (Copyright [24,25,28]).

Among numerous types of optical sensors, which include fluorescence, surface plasmon resonance (SPR), surface-enhanced Raman scattering (SERS), and others, in the field of CK-MB detection, fluorescence sensors are one of the most extensively researched categories. Fluorescent biosensors achieve precise recognition and detection of specific substances by capturing the chemical information of intermolecular interactions and converting this information into changes in fluorescence signals [49]. Wang et al. [50] developed a fluorescent immunoassay method based on boronic acid-modified g-C_3_N_4_ nanosheets (BCNNSs). As illustrated in Figure 4A, this method involved the attachment of boronic acid to g-C_3_N_4_ nanosheets to form Wulff-type boronic acid, which facilitates antibody binding under physiological conditions. During this process, the fluorescence characteristics of g-C_3_N_4_ undergo unique changes: the fluorescence was quenched upon boronic acid attachment, restored upon antibody binding, and quenched again upon the addition of the target antigen. Utilizing this “off-on-off” fluorescence response pattern, sensitive detection of MI biomarkers (cTnI, CK-MB, Myo) was achieved with just a 20 min single-step incubation. For CK-MB detection, the fluorescence intensity exhibited a good correlation with concentration, with a linear range of 0.02–6 ng/mL and a LOW as low as 74.0 fM, demonstrating high sensitivity and a good linear relationship. This study provided an efficient technical means for rapid clinical detection. Additionally, immunochromatographic test strips (ICTSs) were also commonly used for screening AMI biomarkers. Yuan et al. [51] integrated a self-driven microfluidic chip with fluorescence immunoassay for multi-target detection of Myo, CK-MB, and cTnI. The chip utilized an ultrasonic spray coating to create a carboxyl-functionalized surface, enhancing biofunctionality, biocompatibility, and antibody probe immobilization efficiency. It was divided into four collaborative modules. Fluids moved via capillary forces: the sampling zone (Module a) introduced samples; the reaction zone (Module b) regulated flow and mixing through regions with distinct capillary forces, ensuring full sample–probe contact; the timing control valve (Module c) used dense grooves to reduce flow rate, prolong mixing, and guide fluid to the detection zone; and the detection zone (Module d), with over 11,000 microstructures, increased reaction surface area and antigen capture efficiency, improving detection range and sensitivity for efficient biomarker detection. This method showed excellent CK-MB detection performance, with a linear range of 1 ng/mL to 70 ng/mL and a detection time of 10–15 min. Lai et al. [52] developed a fluorescent immunochromatographic assay (FL-ICA) based on europium (III) chelate-modified polystyrene microparticles (CM-EUs) for the rapid, sensitive, and quantitative detection of CK-MB in serum samples. The carboxylic acid-based microparticles modified with europium (III) chelates could covalently bind to proteins, which not only enhanced the stability of the fluorescent labeling but also reduced interfering factors. During the actual detection process, the primary antibody against CK-MB is first immobilized on the test line, and the secondary antibody against CK-MB conjugated with europium (III) chelate microparticles is placed in the conjugate pad of the test strip. CK-MB in the sample pad flows towards the conjugate pad by capillary action and binds to the secondary antibody. It then continues to migrate along the membrane until it reaches the test line, where it binds to the primary antibody against CK-MB. At this point, the concentration of CK-MB is quantitatively analyzed by detecting the fluorescent signal. Rabbit IgG was also used as a quality control group in the experiment. The test strip exhibits a linear range of 0.85–100.29 ng/mL for CK-MB detection, with a LOD of 0.029 ng/mL. It also demonstrated good repeatability and recovery rates, providing a simple, rapid, sensitive, and reliable method for the point-of-care detection of CK-MB concentration in serum. As illustrated in Figure 4B, Gong et al. [53] designed a rapid immunoassay platform using MNPs’ fluorescence quenching effect on Cy5. They integrated MNP probes and Cy5 into an immunochromatographic strip with dual fluorescent and colorimetric readouts. Under a bright field, MNP aggregation at the T-line produced a colorimetric signal for quick qualitative detection. In a dark field, MNPs quenched Cy5 fluorescence at the T-line for sensitive quantitative analysis. This method enabled simultaneous qualitative and quantitative detection of cTnI and CK-MB. Cy5-labeled antibodies for CK-MB and cTnI were placed on two test lines, while MNP probes with detection antibodies were sprayed on conjugate pads. Target analytes triggered sandwich immunoassays, generating colorimetric signals and quenching fluorescence for sensitive detection. The assay showed high sensitivity (LOD: 0.085 ng/mL for CK-MB, 0.049 ng/mL for cTnI), good selectivity, and stability, matching electrochemiluminescence results in 30 clinical samples.

In addition to the aforementioned fluorescent immunosensors, aptamer-based fluorescent biosensors have also demonstrated significant potential and value. As illustrated in Figure 4C, Jiang et al. [54] presented a signal-amplified sandwich aptamer-based visual lateral flow assay (bFNA-LFA) using self-assembled bifunctional nucleic acids (bFNAs).The bFNA is constructed via hybridization chain reaction (HCR), where an initiator probe opens H1 and H2 hairpins to form double-stranded DNA, with functional arms binding AuNPs@DNA and aptamer (Hapt.21). This pre-assembly replaces gold nanoparticles in traditional LFAs, saving time and simplifying operations. For detection, bFNA captures CK-MB to form a complex that flows to the test line, generating a red signal via Apt.30. Without CK-MB, no signal appears. Excess bFNA is captured on the control line for validation. This method enhances sensitivity without increasing time, achieving a 7.9 ng/mL detection limit and 100–1500 ng/mL linear range in 15 min. Zhang et al. [55] developed a fluorescent microsphere LFA (FL-LFA) for CK-MB detection using magnetic bead-screened aptamers. Traditional colloidal gold LFA lacks quantification accuracy and needs better sensitivity. Fluorescent microspheres enable both qualitative and quantitative analysis due to their stable properties and modifiable surfaces. Aptamers C.Apt.21 and C.Apt.30 (with low dissociation constants) were selected via SELEX. C.Apt.21 was fixed on the test line, its complementary DNA on the control line, and C.Apt.30, conjugated with fluorescent microspheres, on the sample pad. In the presence of CK-MB, a sandwich structure forms at the test line, generating fluorescent signals on both lines. Without CK-MB, only the control line shows fluorescence. This method achieves a LOD of 0.63 ng/mL and a range of 0.005–2.0 μg/mL, unaffected by other AMI biomarkers, with serum recovery rates of 88–117%, providing an accurate CK-MB detection option.

In summary, fluorescence-based biosensors have demonstrated significant potential for CK-MB detection, offering high sensitivity (with detection limits reaching the fg/mL level), rapid analysis times (often under 20 min), and the capacity for multiplexed detection. Despite their strong clinical applicability, stability remains a key challenge. Environmental factors such as pH, temperature, and humidity can adversely affect nanomaterial modifications and the reproducibility of the fluorescence signal. The long-term storage of fluorescent labels also poses a risk of degradation, aggregation, or leakage. Furthermore, complex sample matrices can lead to non-specific adsorption and signal deviations. The intricate preparation processes required for some systems, including aptamer screening and nanomaterial functionalization, also present barriers to large-scale manufacturing and clinical translation. Future research must focus on optimizing material stability, enhancing environmental adaptability, and improving resistance to matrix interference.

**Figure 5:**
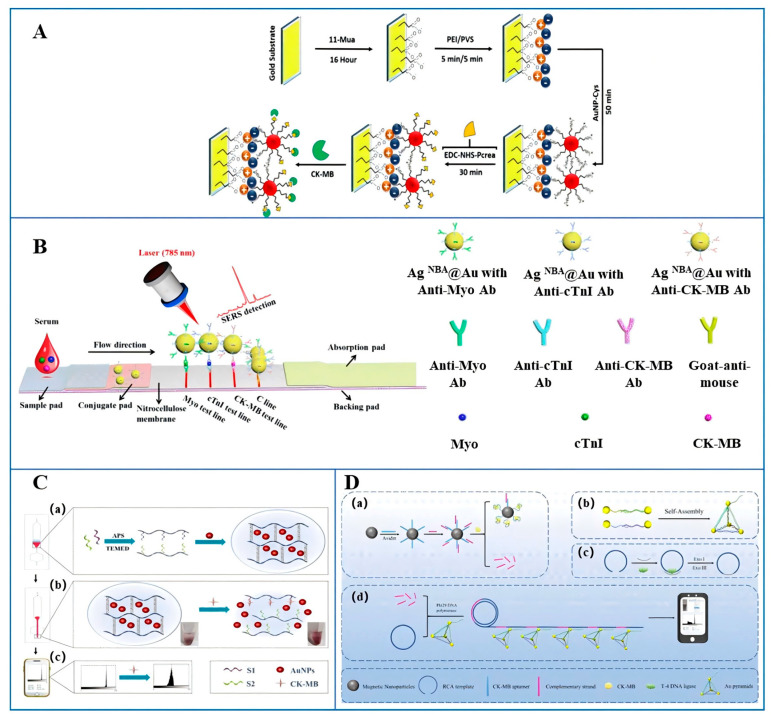
Other optical biosensors for the detection of CK-MB. (**A**) An SPR-based optical biosensor [58]; (**B**) a SERS-based optical biosensor [60]; (**C**) a DNA hydrogel-based optical biosensor. (**a**) At the inlet zone of the microfluidic chip, AuNPs-encapsulated hydrogel was prepared with S1 and complementary short strand S2, (**b**) After the introduction of CK-MB, S1 could dissociate and destabilize the hydrogel to release encapsulated AuNPs, (**c**) The subsequent result could be monitored by naked eye and average grayscale value changes can be obtained by ImageJ [62]; (**D**) an RCA-based optical biosensor. (**a**) MNPs-aptamer probes competitively detected the target to release cDNA, (**b**) the formation of the Au tetrahedron, (**c**) the formation of the RCA template, (**d**) RCA-Au tetrahedron complexes were formed, and the signal was read by ImageJ [64] (Copyright [32,34,36,38]).

In other optical biosensors, various physical phenomena of light, such as absorption, reflection, refraction, interference, and scattering, exhibit changes that are closely related to the properties of the measured substance. In the detection of CK-MB, the reported optical sensors include SPR and SERS. SPR technology, as a simple and direct sensing technique, measures the changes in refractive index near the metal surface to investigate the properties of the studied substances [56]. As illustrated in Figure 5A, Kim et al. [57] addressed the high cost of traditional SPR sensor chips and the susceptibility of antibody activity during the regeneration process by innovatively introducing an “ionic calcium switch” mechanism, constructing a regenerable SPR-based immunosensor. By utilizing a monoclonal antibody sensitive to conformational changes in calcium-binding protein (CBP) and immobilizing it on the sensor surface. combined with CBP and the capture antibody complex targeting CK-MB, a detection system was constructed in the presence of calcium ions. After detection, the calcium ions were removed to regenerate the sensor surface, followed by subsequent detection with fresh complexes. This method successfully achieved semi-continuous real-time monitoring of CK-MB, with a LOD of 2.71 ng/mL. It exhibited a good linear relationship within the concentration range of 1.75–500.75 ng/mL and remained stable over a period of up to one month, providing an economical and reliable solution for clinical repeated detection of CK-MB. Ferreira et al. [58] employed a layer-by-layer self-assembly technique to modify the electrode surface with cysteamine-stabilized gold nanoparticles (AuNPs) and creatine phosphate, constructing a novel CK-MB biosensor that significantly enhances detection sensitivity. In their experiment, cysteine (Cys)-stabilized AuNPs were used and functionalized with creatine phosphate, effectively improving the sensitivity and accuracy of CK-MB detection in saliva and urine samples. Through a series of treatments, the gold nanoparticle–cysteine (AuNP-Cys) and activated creatine phosphate were immobilized on the substrate surface. The interaction between CK-MB and creatine phosphate enabled the SPR technique to detect subtle changes in the refractive index on the metal surface, thereby achieving quantitative detection of CK-MB. This sensor exhibited a detection limit of 0.209 ng/mL for CK-MB and a sensitivity range of 5.0–100.0 ng/mL, demonstrating excellent detection performance.

SERS analysis is an analytical method rooted in Raman spectroscopy technology, which significantly enhances the Raman scattering signal intensity of adsorbed molecules through the surface enhancement effect on specially prepared metal conductor surfaces or within sols [59]. Zhang et al. [60] proposed a LFA method using core–shell SERS nanotags. As shown in Figure 5B, Raman dyes were embedded in silver-core gold-shell nanoparticles (AgNBA@Au) to create SERS nanotags for detecting three AMI biomarkers (Myo, cTnI, CK-MB). Antibodies for these biomarkers were immobilized on three test lines, and core–shell nanoparticles with secondary antibodies were placed on the binding pad. The sample moved via capillary action, forming antibody sandwich structures on the test lines. Target presence was confirmed by color change and SERS signal intensity. Quantitative detection relied on SERS signals from AgNBA@Au nanoparticles. The LOD for CK-MB was 0.55 pg/mL, with a wide linear range (0.02–90 ng/mL) and good reproducibility (8.5% CV), correlating well with clinical chemiluminescent immunoassay results. Cheng et al. [61] constructed a SERS-based immunoassay platform utilizing gold pattern array chips and Au@Ag core–shell nanoparticles for the detection of cTnI and CK-MB. To capture the target antigens, monoclonal antibodies specific to these two biomarkers were immobilized on the surface of the gold substrate. Subsequently, polyclonal antibody-conjugated Au@Ag core–shell SERS probes were added to form sandwich immunocomplexes. Quantitative analysis of these biomarkers could then be performed by monitoring the characteristic Raman peak intensities of the Raman reporter molecules attached to the SERS probes. The platform achieved a LOD of 9.7 pg/mL for CK-MB. Additionally, recovery experiments were conducted in normal human serum samples, with the recovery rates for spiked CK-MB serum samples ranging from 97.3% to 113.2%.

In addition to the aforementioned relatively common optical biosensors, the detection strategy based on DNA hydrogels has brought new breakthroughs to the detection of CK-MB. As illustrated in Figure 5C, Chen et al. [62] integrated target-responsive DNA hydrogels with a microfluidic chip. The CK-MB aptamer and a complementary short DNA strand were grafted onto polyacrylamide chains to form the hydrogel. In the presence of CK-MB, the aptamer bonds to it, causing the hydrogel to dissociate and release pre-captured gold nanoparticles. By analyzing the grayscale values generated from the color change of the solution through mobile phone photography, portable and visual quantitative detection of CK-MB was achieved, with a LOD of 0.027 nM. This method was simple to operate and highly portable, making it particularly suitable for on-site detection scenarios with limited resources and offering new options for point-of-care testing. Notably, the team further constructed a programmable non-nucleic acid target detection platform that leverages the collateral cleavage activity of Cas14a to alter the mechanical properties of DNA hydrogels [63]. By using magnetic beads conjugated with aptamers, complementary DNA (cDNA) is competitively released in the presence of CK-MB. The cDNA underwent exponential amplification reaction (EXPAR) and subsequently activated CRISPR/Cas14a, which cleaved the cross-linking chains of the DNA hydrogel, releasing encapsulated PtNPs/Cu-TCPP(Fe). Through catalyzing the reaction of 3,3′,5,5′-tetramethylbenzidine (TMB), quantitative detection of CK-MB was achieved by measuring the absorbance at 450 nm. This system achieved a detection limit of 0.355 pM for CK-MB, with a linear range of 5 × 10^−4^ to 100 nM.

Moreover, Zhang et al. [64] proposed a dual-sensitized smartphone-based colorimetric strategy utilizing rolling circle amplification (RCA) coil-aggregated gold tetrahedra for the detection of CK-MB. The target competes with the aptamer for binding to the cDNA, and the released cDNA serves as a primer for RCA, generating a large amount of single-stranded DNA (ssDNA) and achieving the first signal amplification. The gold tetrahedron, formed by the hybridization and folding of four DNA strands modified with Au, could aggregate more AuNPs and further amplify the signal upon binding to the RCA product. Through the mutual recognition between the gold tetrahedron and the RCA product, an RCA-Au tetrahedron complex was formed, thereby changing the color of the solution. To further enhance the portability of the biosensor, the RCA-Au tetrahedron strategy was integrated with a smartphone, and the grayscale values of the solution were analyzed, enabling quantitative detection of CK-MB. This system achieved a LOD of 0.8 pM and a linear range of 1 × 10^−4^ to 50 nM. This strategy exhibited good specificity and portability, requiring no large-scale instrumentation, and enabled real-time detection of CK-MB under complex conditions, providing a new detection method for biosensing and clinical diagnosis. Huang et al. [65] developed an optical biomolecular detection device based on reduced graphene oxide (rGO). This device innovatively incorporated a silver deposition signal amplification technique, utilizing an alkaline phosphatase (ALP)-labeled secondary antibody to catalyze a redox cycling enzyme-promoted silver deposition reaction. In the detection of CK-MB, the CK-MB in the sample forms a sandwich structure with the capture antibody and the detection antibody. The ALP on the secondary antibody catalyzes the substrate reaction, promoting silver deposition on the rGO surface and thereby enhancing the detection signal. This device achieved a minimum detection concentration of 0.1 ng/mL for CK-MB, with a near-linear relationship in the concentration range of 0.1–10 ng/mL, and also demonstrated good selectivity. Additionally, the rGO-based optical detection device offered advantages such as wide applicability, deep detection capability, and no need for adjusting the incident angle, opening up new directions in the field of optical biomolecular detection.

In summary, advanced optical detection techniques offer precise, simple, and accurate methods for detecting cardiac biomarkers at clinically relevant concentrations, facilitating the early diagnosis of cardiovascular diseases. However, these optical biosensors have inherent drawbacks that can impede their clinical translation. SPR systems, for example, rely on high-precision optical components, leading to high instrumentation costs, and the long-term stability of regenerated sensor chips requires further validation. SERS faces challenges related to the complex and costly synthesis of nano-enhanced substrates, and its signal is susceptible to environmental interference (e.g., pH, temperature), resulting in batch-to-batch variability. DNA hydrogels are prone to degradation during long-term storage, and environmental humidity can cause non-specific dissociation. Finally, techniques like RCA involve multi-step enzymatic reactions, making the process cumbersome and susceptible to contamination. Overcoming these limitations through material optimization, process simplification, and enhanced anti-interference designs is essential for their successful clinical implementation.

**Table 1:** A Summary of CK-MB detection using different biosensors.

Type of Biosensor	Recognition Elements	LOD (ng/mL)	Liner Range (ng/mL)	Type of Sample	Recovery Rate (%)	Ref.
Electrochemical	Antibody	0.16	0.5~2000	Human Serum	-	[33]
Electrochemical	Antibody	0.00088	0.001~2000	Human Serum	98.60~101.20	[34]
Electrochemical	Antibody	0.26	5~100	Urine	105.92~115.10	[35]
Electrochemical	Aptamer	0.0024	0.01~100	Human Serum	-	[39]
Electrochemical	enzyme	110	190~2880	Human Serum	-	[40]
CL	Antibody	0.296	0.01~500	Human Serum	90.00~110.00	[44]
CL	Antibody	0.067	0.2~120	Human Serum	92.00~106.40	[45]
CL	Antibody	1.37	0.08~10.24	Human Serum	-	[46]
ECL	Antibody	0.000005	0.00005~10	Human Serum	98.00~103.00	[48]
Fluorescence	Antibody	0.003182	0.02~6	Human Serum	-	[50]
FL-ICA	Antibody	1	1~70	Human Serum	-	[51]
FL-ICA	Antibody	0.029	0.85~100.29	Human Serum	90.17~112.63	[52]
FL-ICA	Antibody	0.089	0.02~10	Human Serum	-	[53]
FL-LFA	Aptamer	7.9	100~1500	Human Serum	96~108.67	[54]
FL-LFA	Aptamer	0.63	5~2000	Human Serum	88.00~117.00	[55]
SPR	Antibody	2.71	1.75~500.75	Human Serum	-	[57]
SPR	Antibody	0.209	5~100	Saliva	98.11~107.50	[58]
SERS	Antibody	0.0005	0.02~90	Human Serum	-	[60]
SERS	Antibody	0.0097	-	Human Serum	97.30~113.20	[61]
DNA hydrogel	Aptamer	1.161	8.6~26,875	Human Serum	96.63~106.25	[62]
DNA hydrogel	Aptamer	0.016	0.0215~4300	Human Serum	97.93~106.24	[63]
RCA	Aptamer	0.07	0.0043~2150	Human Serum	92.00~107.00	[64]
RGO	Antibody	0.1	0.1~10	Human Serum	-	[65]

## 3. Conclusions and Prospects

Recent advancements in biosensing technology have made significant breakthroughs in the early diagnosis and prognostic management of AMI through the enhanced detection of CK-MB. Characterized by high sensitivity, wide linear ranges, and rapid response times, these technologies are progressively reshaping the clinical diagnostic landscape for cardiovascular diseases. Continuous innovation across diverse platforms—including electrochemical, chemiluminescent, fluorescent, and optical sensors—is driving CK-MB detection toward greater precision. Notably, aptamer- and immunoassay-based biosensors have demonstrated exceptional specificity and sensitivity, while emerging technologies such as fluorescent immunochromatography and DNA hydrogels offer distinct advantages in portability and multimodal detection.

The foundational strengths of these biosensors arise from the synergistic integration of materials science, advanced optical detection, and system engineering. The strategic modification of sensor surfaces with nanomaterials, such as polypyrrole@bismuth tungstate heterojunctions and ultrathin AuPdCu alloys, has proven critical in enhancing electron transfer efficiency and catalytic activity. In parallel, optical technologies like SERS and SPR facilitate label-free, highly sensitive, and real-time monitoring. The convergence of microfluidics with smartphone-based platforms, exemplified by innovations like 3D-printed functional microspheres, is fostering the development of portable and intelligent POCT devices. These advancements are particularly crucial for providing feasible and accessible AMI screening solutions in resource-limited settings.

Despite these promising developments, the clinical translation of advanced biosensing technologies is impeded by several significant challenges. A primary limitation is that most platforms have been validated only at the laboratory scale or with limited sample sizes; large-scale, multicenter clinical trials are required to corroborate their efficacy and reliability in real-world settings. The inherent complexity of biological matrices, such as whole blood and saliva, presents another major obstacle, as matrix effects can interfere with detection accuracy. This necessitates the development of more effective sample pretreatment methods or the design of more robust, anti-fouling sensing interfaces. Furthermore, issues related to standardization, including batch-to-batch variability in nanomaterials, the long-term storage stability of biorecognition elements, and sensor reusability, currently hinder widespread adoption. Key technical hurdles, such as signal cross-interference and dynamic range matching in multiplexed biomarker detection, also remain critical areas for future research.

In the future, the evolution of CK-MB biosensor technology is poised for multidimensional innovation. The fusion of electrochemical, optical, and microfluidic systems with artificial intelligence, machine learning, and the Internet of Things (IoT) will be instrumental in creating fully integrated, intelligent diagnostic platforms. Such systems could enable automated “sample-in-result-out” operations, complete with self-calibration and cloud-based data analytics. The development of novel recognition elements and materials, including molecularly imprinted polymers and biomimetic nanostructures, promises to enhance sensor stability, environmental tolerance, and anti-interference capabilities. Advanced manufacturing techniques, such as 3D and inkjet printing, will facilitate the scalable production of flexible electrodes and wearable sensors, enabling continuous, real-time CK-MB monitoring. Moreover, integrating multi-omics data will allow for the construction of sophisticated AMI risk prediction models, transitioning the diagnostic paradigm from single-marker detection to holistic patient management. To realize this potential, establishing interdisciplinary collaborations and formulating clear industry standards will be essential to accelerate the translation of laboratory research into clinically approved products, such as POCT kits, and to overcome bottlenecks in the industrial-scale manufacturing of micro- and nano-structured devices.

In summary, biosensing technology is catalyzing a paradigm shift in cardiovascular diagnostics through the deep integration of advanced materials, novel sensing modalities, and integrated systems. As research continues to push the frontiers of science and engineering, the emergence of next-generation CK-MB detection systems, which are characterized by ultrahigh sensitivity, multiplexing capabilities, and clinical practicality, is on the horizon. These innovations are poised to provide clinicians with more precise and timely diagnostic tools, ultimately contributing to reduced AMI mortality, improved patient outcomes, and an elevated standard of care in cardiovascular disease management.

## Data Availability

Not applicable.
